# A mixed method study into obstetric sonographer-led-discharge and other forms of sonographer role extension

**DOI:** 10.1177/1742271X211038296

**Published:** 2021-09-13

**Authors:** LS Kettlewell, SP Richards

**Affiliations:** 1 James Cook University Hospital, Middlesbrough, UK; 2 Department of Allied Health Professions, The School of Health & Life Science, Teesside University, Middlesbrough, UK

**Keywords:** Advanced practice, career progression framework, role development, sonographic career structure

## Abstract

**Introduction:**

Sonographer-led-discharge was proposed in a maternity unit to provide a holistic service, cut waiting times, ease staffing pressures and increase job satisfaction. This study explored sonographers’ experiences and perspectives of this new extended role and other areas of non-obstetric role extension. Understanding these will inform future practice and the success of the proposed obstetric sonographer-led-discharge and career structure.

**Methods:**

A mixed methodology, cross-sectional study was performed, with a purposive, non-probability sample using an online data collection tool. The data were analysed using descriptive statistics and thematic analysis.

**Results:**

In total, 93 sonographers participated in the study. Of these, 25% of sonographers currently practising obstetric ultrasound said they would not undertake the proposed obstetric sonographer-led-discharge role extension although 90% of the participants said role extension provides job satisfaction. Several themes emerged from the data, including job satisfaction, benefits to the hospital, improved patient pathway, time, personal factors, litigation and intra- and interprofessional resistance. A total of 54% of staff currently performing a role extension have experienced either inter- or intraprofessional conflict and only 48.5% said their workload was manageable.

**Conclusions:**

The data collected suggested that, with training and support, the proposed obstetric sonographer-led-discharge role is an appropriate role extension for sonographers. These findings support the premise of the proposed sonographic career structure, although the inter- and intraprofessional resistance identified in the study could form a significant barrier if it is not appropriately considered and managed.

## Introduction

There is strong evidence that fetal growth restriction is the biggest risk factor for stillbirth. Antenatal detection is vital and can reduce stillbirth significantly by giving the option of timely delivery of the baby at risk.^1^,^2^ The *Saving Babies’ Lives Care Bundle* was published in 2016 with the aim of halving the number of stillbirths within the UK by 2025.^3^ A later version of these guidelines *Saving Babies’ Lives Version Two* recognised that implementation of the recommended care bundles has had a significant impact on the capacity of service providers.^4^ This included a rise in the number of referrals sent for an ultrasound scan to assess fetal size and wellbeing. There has been an 18% reduction in stillbirths in England compared to figures in 2010; however, further changes are required to meet the target of 50%.^5^ The number of births in England has risen by a quarter in the last decade.^6^ This, paired with a national NHS staff shortage issue, has added pressure to an already stretched service and therefore healthcare needs to evolve and adapt to meet these demands.^7^

This was demonstrated in a maternity assessment unit (MAU) where role extension was proposed for sonographers working in the unit to address issues such as high waiting times, departmental staffing pressures and a disjointed service. The aims of this role extension were to provide a holistic service for patients, to cut waiting times, ease staffing pressures and increase job satisfaction. Although the national guidance from the Perinatal Institute suggests that a patient referred by a community midwife should have an ultrasound assessment of fetal growth, performed by a sonographer, within 72 hours of referral,^8^ local practice in the MAU at this Trust requires that this assessment is conducted within 48 hours of referral. If complications are detected, the patient must be reviewed by a specialist trainee in obstetrics and gynaecology, year 3 or above, to determine a management plan. In the proposed role extension, the patient would not need to be reviewed and the sonographer would put the care plan in place after undertaking the scan and, if appropriate, discharge the patient. The care pathways the sonographers would follow include guidance on scan outcomes such as low or high estimated fetal weight, abnormal umbilical Doppler/liquor volume or no fetal movements seen.

Sonographers are a diverse group of healthcare professionals who work at an advanced level of practice. Approximately 70% of sonographers are qualified radiographers, although midwives, nurses, doctors and other registered healthcare professionals can also undertake ultrasound training. For some, performing ultrasound examinations and providing an expert opinion will be their fundamental role, while for others ultrasound is used as a tool to assist with a diagnosis, monitor a condition or enhance patient care.^9^ Advanced practitioners (including sonographers) should possess advanced clinical skills, postgraduate level education and provide a role that responds to the needs of the patient.^10^

Some sonographers also undertake role extension traditionally performed by radiologists such as ultrasound-guided biopsies, fine needle aspiration and cytology and patient management, similar to the proposed role extension outlined in this study. These role extensions demonstrate that the role of a sonographer has evolved significantly.

Health Education England is responsible for commissioning university training and forward planning to ensure the future NHS workforce will be available to meet the needs identified in the five-year forward view.^11^,^12^ A vital part of ensuring the workforce is ready and is able to understand the views and needs of the current workforce. This understanding will also assist in employee retention and inform future practice.

To increase the sonographer workforce, the traditional sonographer’s role and training is under review. Proposed changes are to develop undergraduate courses, direct-entry ultrasound training, advanced apprenticeships and a four-tier career structure similar to other allied health professions. This four-tier structure would include a sonographer, senior sonographer, advanced practitioner and consultant practitioner.^12^ Health Education England describe how these differences in training and career pathway could also provide job satisfaction and opportunities for progression, improve retention and increase the capacity of the service. These changes are felt to be necessary to protect the future of ultrasound services.^12^

This study explored sonographers’ experiences and perspectives of an obstetric sonographer-led-discharge role extension, and other areas of non-obstetric role extension. Wider non-obstetric role extension was included as it was unknown if other departments had implemented a similar obstetric sonographer-led-discharge pathway and no previous research was identified proposing a role extension of this type. The authors also wanted to gain an understanding of sonographers’ experiences and perceptions when undertaking wider role extension to understand and inform future practice. Change can be unsuccessful if we do not understand the values, attitudes, thoughts and work culture influencing it. To the best of the authors’ knowledge no similar work has been published.

## Methods

A mixed methodology, cross-sectional study was performed, using an online data collection tool. This methodology was used to collect quantitative data and explore sonographers’ experiences and perceptions on the proposed obstetric sonographer-led-discharge and wider role extension.

Ethical approval for this research was granted by the School of Health and Social Care Committee at Teesside University (Reference: 035/18 on 4 April 2018).

A purposive, non-probability sample consisting of qualified sonographers was used to collect thought-provoking and valuable data. Participants were recruited via The Society and College of Radiographers (SCOR) publication ‘Synergy News’ and the British Medical Ultrasound Society (BMUS) electronic newsletter ‘Ultrapost’. These societies were used as 70% of sonographers in the UK are registered diagnostic radiographers^9^ and BMUS has a wide membership of healthcare professionals practising ultrasound. The inclusion criterion was kept broad to collate as much information as possible; therefore, sonographers from all backgrounds and specialties were eligible to participate. For the purpose of this research, sonographers are healthcare professionals who have a qualification in medical ultrasound, undertake diagnostic ultrasound examinations and provide written reports. Sonography is not a registered profession, sonographers in the UK come from a wide range of backgrounds. In 2017, there were 530 registered sonographers on the voluntary register held by SCOR.^13^ Using this figure, a target sample size for this research was 233 sonographers (95% confidence interval, 5% marginal error). Achieving the target sample size could allow the findings of the research to be generalised to the population of interest.

## Procedures

### Data collection methods

A data collection tool was designed for this study and piloted with a group of 10 sonographers. The tool was accessed via the Bristol Online Survey website. The tool included brief and simplified questions to try and increase the response rate. The collection tool included statements and questions, derived from themes extracted during a literature review performed prior to undertaking this study and relevant policies and legislation. Statements were paired with a Likert scale with a range from strongly agree to strongly disagree. Open-ended questions were included to allow the participant to provide depth to their answers and share their experiences. To ensure participants only answered questions relevant to their practice, the tool was constructed to ensure respondents were directed to the questions that are suitable for them to answer based on previous answers. The tool was open to participants between 14 April and 18 July 2018, with regular electronic reminders (email and social media) sent to request and encourage participation.

### Data analysis

As the mixed methods approach for this research was complementary, the data analysis was undertaken at the interpretation point. Descriptive statistics were used to evaluate the quantitative data. Thematic analysis was used to identify themes in the participants’ answers.

The first step undertaken was the data management stage; this included reading and familiarisation to identify the first group of themes, these developing themes were recorded to identify overlap and recurrence to then filter the data. Significant themes were then identified for further investigation and then all data were sorted into these themes. The coding was undertaken by two coders to reduce bias and improve validity. After data familiarisation, management and initial codes, thematic mindmaps were used to begin exploring the emerging themes. The identification of the themes was initially carried out independently by the coders and once completed, these findings were discussed at length, mutually agreed and overlapping themes identified.^14^

## Results

The following is a discursive analysis of the data, stating the descriptive analysis and exploring themes identifying during the thematic analysis.

### Sample demographics

**Figure 1.**
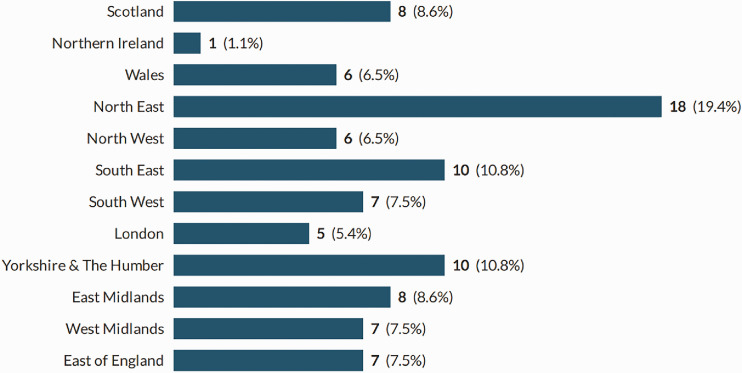
Participant location.

**Figure 2.**
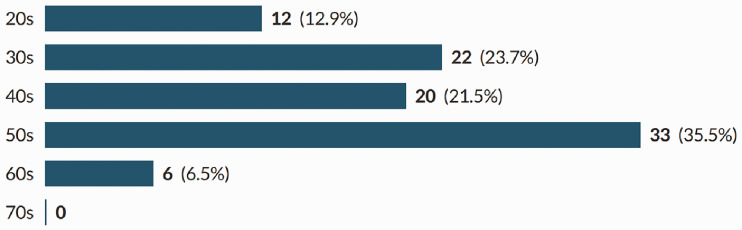
Participant age.

**Figure 3.**
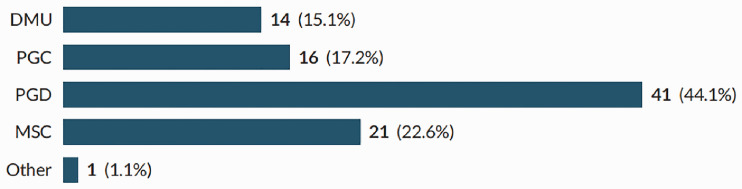
Level of qualification.

**Figure 4.**
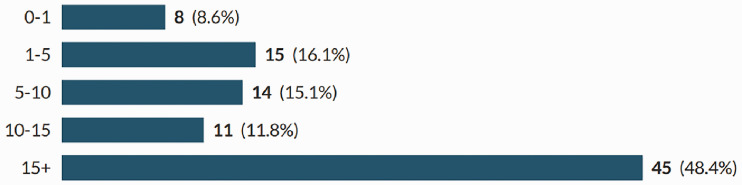
Years from award of ultrasound qualification.

**Figure 5.**
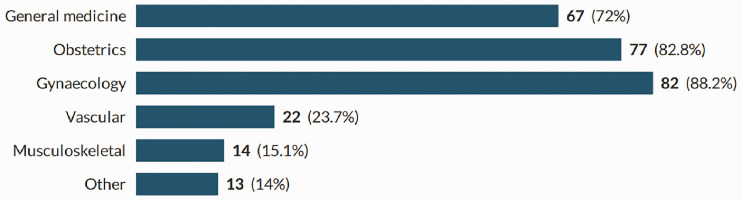
Area of specialty.

**Figure 6.**
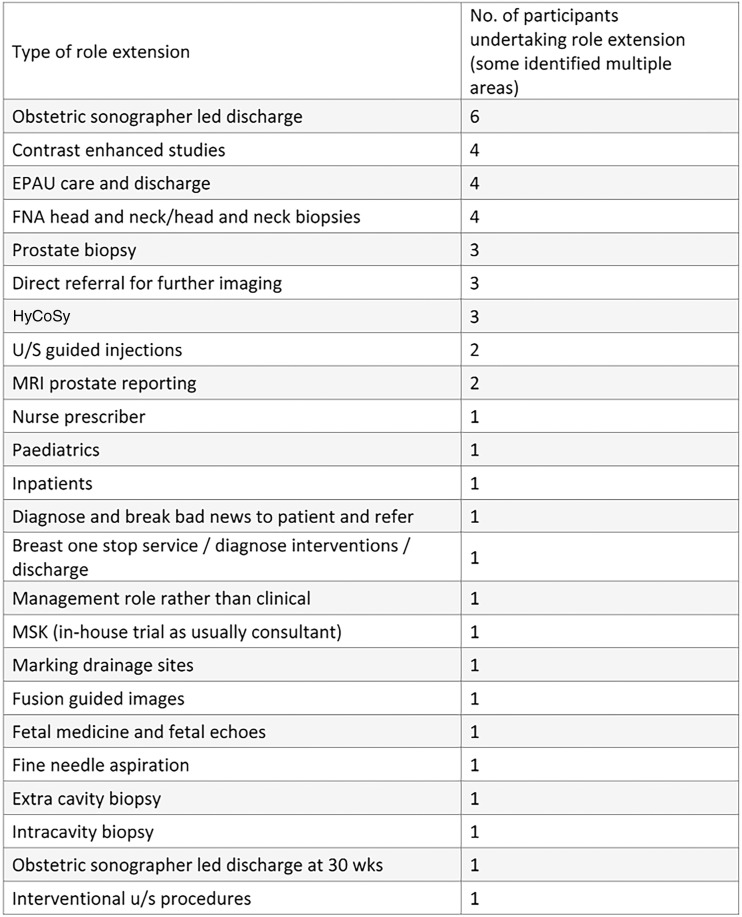
Area of role extension as stated by the participants.

In total, 93 sonographers completed the survey; a response rate of 38%. Participants were recruited from all countries of the UK, across the working age (21–67), with the majority of participants being in their 50s (Figures 1 and 2). The level of qualification varied and included Diploma of Medical Ultrasound, postgraduate certificate, postgraduate diploma (PgD) and Master of Science with one participant stating another qualification (PgD and Society for Vascular Technology). The PgD was the most commonly held qualification (44.1%) (Figure 3). Participants’ experience ranged from newly qualified to over 15 years, with 48.4% being awarded their qualification more than 15 years prior to participating in the study (Figure 4). The sample included sonographers practising across a range of specialties, with most practising in more than one area (Figure 5). Thirty-two participants (34.8%) stated they have an extended role, either obstetric or non-obstetric. Figure 6 lists the areas of role extension as described by the participants themselves; the authors have chosen not to alter their wording. Some participants perform more than one form of role extension.

**Figure 7.**
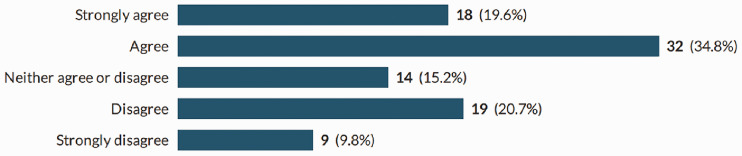
Litigation.

Of all the participants performing a form of role extension, 27.3% stated they had not received any training to undertake their extended practice. The participants undertaking role extension were asked about their experiences and perspectives of performing their role. Only 36.3% believed they were remunerated appropriately. In total, 48.5% felt their workload was manageable, 15% felt they were not supported in their role and 54.5% had encountered interprofessional conflict, although 90% stated that their role extension gave them job satisfaction.

Thirty-two (34.4%) participants stated they currently undertake a form of obstetric sonographer-led-discharge and some did not consider this to be role extension but simply a routine part of their job.

### Participants’ perceptions on the obstetric sonographer-led-discharge role extension

Thirty-two (34.4%) participants already perform obstetric sonographer-led-discharge. Of the remaining 61 participants, 49% said they would not be willing to undertake obstetric sonographer-led-discharge. Further exploration of the reasons for this suggested that some participants felt obstetric sonographer-led-discharge was not part of their clinical competence, within their remit, they were too busy already or simply they do not scan obstetric patients. When those sonographers not performing obstetric examinations were removed, 25% said they would not perform obstetric sonographer-led-discharge. Some stated the role was more suited to a midwife sonographer rather than one with a background in radiography.

*If you are a midwife sonographer, then this may be appropriate, but just having a normal scan result does not mean everything is all OK.*

*There is much more to obstetrics care than is known by a sonographer.*

Some felt sonographers do not have the time available to complete extra responsibilities and training requirements, and limited staffing would be a barrier to undertaking obstetric sonographer-led-discharge.

*We already take enough responsibility without adding to it.*

*Too far out of my scope of experience/practice. I’d be too nervous to make those decisions.*

The majority of participants (58%) felt that in-house training would be sufficient for obstetric sonographer-led-discharge roles, whereas postgraduate training was considered as essential by 33% of respondents. The following comments suggest that a blended approach to learning is important, utilising a formal training programme with work experience to provide competence for the role extension.

*A mix of post-grad course and spending time with doctors/midwives/CNS as appropriate*

*Certificated short course and evidence of ongoing CPD*

### Thematic analysis of factors related to the proposed sonographer-led-discharge role extension and wider non-obstetric role extension

#### Themes identified influencing role extension

**Increased job satisfaction.** There were many positive aspects identified when describing the advantages of the role extension including adjectives such as rewarding, motivating and providing more confidence. Some participants felt that having more responsibilities as part of a role extension could be progressive and was not perceived as a negative. The participant comments also noted that role extension could provide new challenges, facilitate continuing professional development (CPD), offer very good career development and an extended scope of practice. It was stated that increasing the sonographer’s knowledge by undertaking a role extension has the ability to benefit others, as the knowledge can be passed on to junior staff during mentoring.

**Resistance.** Resistance emerged from the data in three ways: resistance from sonographers who do not want to undertake role extension, resistance from other professionals and resistance from those that do not recognise the role extension.

*Initially there was conflict with the midwifery team but I feel that our team of sonographers have proved ourselves to be competent and also we have integrated more midwives into our scan team so I feel we have built more bridges and are working together better*

More than half (54.5%) of those who perform role extension have experienced interprofessional conflict as part of that role. Professional encroachment concerns and protectionism of the perceived role were described in the responses.

*I am paid as a consultant practitioner however I am not allowed to use the title due to a group of radiologists disagreement with non-medical consultant roles*

*Glass ceiling: some but not all radiologists [are] protective and resist encroachment into their patch*

*There is role extension for nurses and midwives into ultrasound, but it never goes the other way. Why can’t sonographers extend practice in early pregnancy units, for example? At least discharging live intrauterine pregnancies.*

*I come from a radiology background. Midwives expressed concern that radiographer sonographers are not equipped with the people skills required to discuss and empathise with women suffering miscarriage or other early pregnancy complications. Interestingly my ability to diagnose was not called into question, nor was my ability to assess women for sepsis/UTI and other potentially life-threatening early pregnancy complications. Yet I felt well equipped to empathise but worried over missing signs of infection etc.*

*Midwives who train for 3 years currently do this job. I do not agree that a sonographer could pick up this skill very quickly and be sufficiently competent.*

*I do believe that sonographers are capable of role extension for the benefit of both the patients & service demands if only we were given more support. I don't necessarily believe we need just more sonographers but we need to be identifying means by which to motivate & retain those we have which I believe can be partly achieved through role extension opportunities.*

*The fact xx already undertake role extension and clear job satisfaction there must be opportunities to progress*

Some participants have had a positive experience and received a great amount of support from the radiologists and their teams.

*Our team has excellent opportunity for role development. This is supported by radiologists, radiology management and fellow sonographers.*

**Benefits to the hospital and patient pathway.** Participants suggested that extended roles saved time for doctors, helped to overcome the issue of radiologist shortages, reduced pressures on maternity staff and helped facilitate the seven-day service provision. Any intervention that saves time would be beneficial as staff shortages in healthcare are a widespread problem. It also emerged from the data that undertaking role extensions could provide an improvement to interprofessional communication. Participants stated that this obstetric sonographer-led-discharge pathway, along with other role extensions, increased service provision and improved the patient experience.

**Time.** Time was a common theme identified in the data. The benefits of role extension were noted as helping to save time for doctors and less waiting for patients; however, the disadvantages of time are seen from a sonographer’s perspective. To save time for doctors and patients means that more time needs to be allocated to the sonographer. The comments by the participants suggested it would take extra time for training to learn how to deal with complications and more time would be spent on complex cases. Only 54.5% of participants either agreed or strongly agreed that they were allocated enough time to undertake their role extension. Only 48.5% felt their workload was manageable. Considering the qualitative comments, workload influenced the sonographers’ resistance to performing the proposed sonographer-led-discharge.

*There is currently a shortage of sonographers and not enough time in the day to scan patients let alone be involved in their discharge, something we are not trained for. It just adds to the pressure and responsibility we are already under. A definite NO from me.*

**Personal.** It is important to understand the personal factors that could influence a sonographer’s willingness to undertake a role extension. Salary was identified as a personal factor. The comments suggest that those who feel they were paid the correct salary for their level of responsibility were graded at band 8. However, the suggested direct entry or apprenticeship route would see some sonographers working at band 5 or 6 unless they are working at an advanced or consultant practitioner level,^15^ which could include undertaking role extension such as this sonographer-led-discharge service.

**Litigation.** Over half of the sample either agree or strongly agree that the possibility of litigation would affect their decision to undertake role extension (Figure 7).

*Litigation: It is a concern - but it can happen in a ‘normal’ scan.*

*Yes, feel pressurised to do the scans but worry about informal training and litigation.*

*Radiologists/obstetricians are much less likely to provide support to allied healthcare professionals than 10 years ago.*

## Discussion and implications for policy

The obstetric sonographer-led-discharge role extension, outlined in this study was proposed to relieve pressure on busy clinicians, enhance the patient experience and increase job satisfaction. Three quarters (75%) of participants practising obstetric ultrasound, who do not already undertake obstetric sonographer-led-discharge, said they would be happy to perform this form of role extension and the benefits to the hospital and patients were clearly identified in the collected data. A quarter (25%) of participants stated they would not participate in the proposed sonographer-led-discharge service. The reasons for this were multifactorial and the comments provided included time pressures, already having a range of responsibilities, a lack of clinical obstetric knowledge, a belief that midwives were better suited to this role or a nervousness about decision making in this area.

This study identified a clear link (90.7%) between role extension and job satisfaction, and this confirms the findings of other authors.^16^ These findings support the premise of the proposed sonographic career structure with a clear progression from practitioner to consultant practitioner for those who want the extra responsibility associated with each role. Finding a person’s comfortable role will help to retain a happy workforce. Progression through the career levels will naturally require role extension. Any advancement through these levels will need to be fair, clear and transparent. However, the interprofessional resistance identified in this study could be a significant barrier to the successful implementation of the career structure. It is not acceptable to prevent an individual from using a title they have earned or to say a profession does not have the correct ‘people skills’. Role extension has been successful for some professional groups^17^ and this should be available to all professions, not just a few as seems to be the case from participant comments. Thirty-two (34.8%) participants already perform significant role extensions, and another 75% of the sonographers who do not already undertake sonographer-led-discharge state they are willing to undertake this form of role extension. Whatever the sonographic career has in its future, the opportunities must be in place to keep all the workforce ‘satisfied’.

For role extension to be successful, sonographers need to be given the appropriate amount of time to undertake the task, although only 48.5% of the participants already performing a role extension stated that their workload was manageable with workload given as a reason why some participants would not want to undertake the proposed obstetric sonographer-led-discharge service. The participants identified that several training options could be used to facilitate role extension, ranging from in-house to postgraduate university courses. Worryingly, 27.3% of participants stated they had not had training to undertake their extended role.

A person’s salary is a contentious issue, but it is important to note that generally only those being paid at band 8 felt they were appropriately remunerated for their role extension; therefore, work crossing tiers of pay grades should be avoided to prevent job dissatisfaction.

## Strengths, weaknesses and recommendations for future research

This study has provided an overview of the attitudes of sonographers on undertaking obstetric sonographer-led-discharge, or other role extensions. No similar research in this area was identified and therefore no direct comparisons or arguments can be made. This research is exploratory and gives introductory information in this topic to build upon.

The number of respondents is a limitation. The target sample size was not reached and therefore the findings cannot be fully generalised; however, the respondents have identified similar themes related to their practice. This study has a similar response rate to the closest comparison, which provided a good insight into radiographic advanced practice.^16^ To improve response rate, the authors could have considered extending the length of time the survey was available; however, several electronic reminders were sent and the authors noted that the majority of responses were received at the early stages of data collection. Another option may have been to send paper versions of the questionnaire to clinical departments, but this was not possible due to financial constraints.

As time and workload was identified as a theme, perhaps it is not surprising that some potential respondents were not able to participate. As significant barriers to the implementation of role extension have been identified, a follow-up study focusing solely on sonographer experiences and perceptions on role extension with the support of professional bodies would be advised. A strength of the data collected is that participants were recruited from all regions of the UK across a range of professions and specialisms. The majority of respondents were from England; however, the number of respondents from each home country closely correlated to its per capita population.^18^ Some of the data have demonstrated mixed opinions on whether obstetric sonographer-led-discharge is suitable for those with a background in radiography.

A weakness of the study is that we did not collect data on the participant’s clinical background, as inter- and intraprofessional resistance was a major theme identified. Future research exploring attitudes of sonographers should consider focusing specifically on each separate group of professionals. This would allow a greater understanding of those that have had varying training and work experience prior to their ultrasound qualification and how that affects their perception of role extension and why participants have experienced significant inter- and intraprofessional resistance.

## Conclusion

This study used a mixed methodology to assess the attitudes of sonographers on undertaking obstetric sonographer-led-discharge and to gain an understanding of the other role extensions performed by sonographers in the UK. The data collected proved that, with training and support, the proposed sonographer-led-discharge pathway is appropriate and is similar to other types of role extension being performed already. These findings support the premise of the proposed sonographic career structure, but the resistance identified in the study will form a significant barrier if it is not appropriately considered and managed. There must be enough development opportunities for all sonographers to keep the workforce satisfied. A pyramid style career structure with consultant practitioner at the top may struggle to provide these opportunities for role extension, vital for job satisfaction, leading to retention of staff, and career progression. Sonographers who undertake role extension also need to be appropriately remunerated, have a manageable workload and given the training to successfully undertake their role and safeguard the patient.
